# Amino acid substitutions in gyrA and parC associated with quinolone resistance in nalidixic acid-resistant Salmonella isolates

**DOI:** 10.1186/2046-0481-66-23

**Published:** 2013-11-15

**Authors:** Dong Hwa Bae, Ho Jeong Baek, So Jeong Jeong, Young Ju Lee

**Affiliations:** 1 College of Veterinary Medicine, Kyungpook National University, Daegu 702-701, Republic of Korea

**Keywords:** gyrA, Nalidixic acid resistance, *Salmonella*

## Abstract

This study was undertaken to identify and characterize amino acid substitutions in *gyrA* and *parC* related with quinolone resistance of 27 nalidixic acid-resistant (Na^R^) *Salmonella* isolates collected in poultry slaughterhouses in Korea. A total of 51 *Salmonella* isolates were detected from 44.8% (47/105) of the total samples from 15 poultry slaughterhouses examined, among which 27 (52.9%) Na^R^ isolates were detected while ciprofloxacin (Cip) resistance was not present in the isolates. These 27 Na^R^ isolates of DNA sequencing revealed that it contained three types of *gyrA* mutations in only D87 codon. Mutations in the D87 codon resulted in substitutions to G in most of the isolates, but D87Y and D87N exchanges were also detected. Although Cip resistance was absent, reduced susceptibility characterized by mutations in *gyrA* was apparent among *Salmonella* isolates from poultry slaughterhouses in Korea.

## Background

*Salmonellae* are Gram-negative bacteria that are found worldwide in both cold- and warm-blooded animals as well as the environment. These bacteria are the main cause of salmonellosis in mammals and birds. Quinolone resistance in *Salmonellae* has developed over the last three decades since the introduction of Na, the first synthetic lone antimicrobial agent [1]. Several mechanisms of quinolone resistance in *Salmonella* spp. have been documented including point mutations in the quinolone resistance-determining region (QRDR) of DNA gyrase (*gyrA* and *gyrB*) or topoisomerase IV (*parC* and *parE*), expression of efflux pumps on the outer membrane, and plasmid-mediated quinolone resistance [1]. The goal of the present study was to identify and characterize *gyrA* and *parC* mutations associated with quinolone resistance in 27 Na^R^*Salmonella* isolates collected from poultry slaughterhouses in South Korea.

## Methods

A total of 51 *Salmonella* isolates were recovered from 105 samples (15 from the first and 15 from the last chilling waters, and 75 from carcasses) collected at two out of nine duck slaughterhouses as well as 13 out of 41 chicken slaughterhouses located in different regions of South Korea. The first chilling water, the last chilling water, and five carcasses from each slaughterhouse were sampled. Bacteria were isolated from the samples according to the standard International Standardization Organization (ISO)-6579 method [2]. Serotyping was performed by slide and tube agglutination using O and H antiserum (Difco, USA) according to the Kauffmann and White scheme [3]. If two colonies showed the same serotypes and antimicrobial resistant patterns, only one colony was randomly chosen for analysis in this study.

Antimicrobial resistance of all 51 *Salmonella* isolates were evaluated using a disc diffusion test with the following discs (Difco): amikacin (An, 30 μg), ampicillin (Amp, 10 μg), chloramphenicol (C, 30 μg), ceftazidime (Caz, 30 μg), cephalothin (Cf, 30 μg), ciprofloxacin (Cip, 5 μg), cefotaxime (Ctx, 30 μg), cefazolin (Cz, 30 μg), cefepime (Fep, 30 μg), cefoxitin (Fox, 30 μg), gentamicin (Gm, 10 μg), imipenem (Imp, 10 μg), kanamycin (K, 30 μg), nalidixic acid (Na, 30 μg), norfloxacin (Nor, 10 μg), streptomycin (S, 10 μg), trimethoprim/sulfamethoxazole (Sxt, 1.25/23.75 μg), and tetracycline (Te, 30 μg). The results were evaluated according to the Clinical and Laboratory Standards Institute (CLSI) guidelines [4]. *Escherichia coli* ATCC 25922 was used as the control strain.

All 51 *Salmonella* isolates from the present study were further tested for amino acid changes in the QRDR, and screened for the presence of the *gyrA* and *parC* genes. Minimal inhibition concentrations (MICs) were determined for two antimicrobials, Na and Cip, belonging to the quinolone and fluoroquinolone classes of antimicrobials, respectively, using an agar dilution method according to CLSI guidelines [4].

DNA was isolated for further molecular studies using a generation capture column kit (Qiagen, Germany) and stored at -70°C before use. Fragments of the *gyrA* (F, 5′-TGTCCGAGATGGCCTGAAGC-3′; R, 5′-TACCGTCATAGTTATCCACG-3′) and *parC* (F, 5′-CTATGCGATGTCAGAGCTGG-3′; R, 5′-TAACAGCAGCTCGGCGTATT-3′) genes containing the QRDR associated with quinolone resistance were amplified by PCR and sequenced as previously described [5,6]. The PCR products were purified and sequenced by Macrogen Inc. (Daejeon, South Korea). The nucleotide sequences were analyzed with the Basic Local Alignment Search Tool (BLAST) on the National Center for Biotechnology Information (NCBI) website (http://blast.ncbi.nlm.nih.gov/Blast.cgi). DNA sequences of the *gyrA* and *parC* genes were compared to those of native *gyrA* DNA (GenBank accession number X78977) and native *parC* DNA (GenBank accession number M68936), respectively.

## Results and discussion

Resistance of the 51 *Salmonella* isolates to 18 antimicrobials is shown in Table 1. Resistance to at least one antimicrobial was found in 74.5% (n = 38) of the isolates while 12 isolates (23.5%) showed multi-drug resistance to three or more classes of drug. Among the *Salmonella* isolates, resistance to Na (52.9%), S (35.3%), and Am and Te (21.6%) was frequently observed. None of the isolates were resistant to An, Cip, Fox, Imp, Nor, or Sxt. Seven out of eight *S.* Typhimurium isolates remained susceptible to all the tested antimicrobials. *S.* Enteritis and *S.* Hadar isolates showed the highest rates of antimicrobial resistance in this study. Nine out of 18 *S.* Enteritidis isolates were resistant to at least three antimicrobial agents while the remaining nine isolates were resistant to one antimicrobial compound. All five *S*. Hadar isolates were resistant to more than two antimicrobial agents. A previous report determined that *S.* Hadar is one of the most resistant *Salmonella* serotypes [7].

**Table 1:** **Prevalence of antimicrobial resistance in 51 *Salmonella *** **isolates from poultry slaughterhouses**

Antimicrobials ^*^	**No (%). of serovars**									**Total**
	**Enteritidis**	**Montevideo**	**Typhimurium**	**Hadar**	**London**	**Ohio**	**Newport**	**Senftenberg**	**Hogton**	
	**(n = 18)**	**(n = 9)**	**(n = 8)**	**(n = 5)**	**(n = 3)**	**(n = 3)**	**(n = 2)**	**(n = 2)**	**(n = 1)**	**(n = 51)**
Na	18 (100)	5 (55.6)	-	-	-	-	2 (100)	2 (100)	-	27 (52.9)
S	9 (50.0)	-	1 (12.5)	5 (100)	1 (33.3)	1 (33.3)	-	-	1 (100)	18 (35.3)
Am	8 (44.4)	-	-	2 (40.0)	1 (33.3)	-	-	-	-	11 (21.6)
Te	3 (16.7)	-	1 (12.5)	4 (80.0)	1 (33.3)	1 (33.3)	-	-	1 (100)	11 (21.6)
Cf	3 (16.7)	-	-	2 (40.0)	1 (33.3)	-	-	-	-	6 (11.8)
K	1 (5.6)	2 (22.2)	-	2 (40.0)	-	-	-	-	1 (100)	6 (11.8)
Cz	1 (5.6)	-	-	2 (40.0)	-	-	-	-	-	3 (5.9)
Gm	1 (5.6)	2 (22.2)	-	-	-	-	-	-	-	3 (5.9)
C	1 (5.6)	-	-	-	-	-	-	-	-	1 (2.0)
Caz	1 (5.6)	-	-	-	-	-	-	-	-	1 (2.0)
Ctx	1 (5.6)	-	-	-	-	-	-	-	-	1 (2.0)
Fep	1 (5.6)	-	-	-	-	-	-	-	-	1 (2.0)
An	-	-	-	-	-	-	-	-	-	0 (0.0)
Cip	-	-	-	-	-	-	-	-	-	0 (0.0)
Fox	-	-	-	-	-	-	-	-	-	0 (0.0)
Imp	-	-	-	-	-	-	-	-	-	0 (0.0)
Nor	-	-	-	-	-	-	-	-	-	0 (0.0)
Sxt	-	-	-	-	-	-	-	-	-	0 (0.0)

^*^An, amikacin; Am, ampicillin; C, chloramphenicol; Caz, ceftazidime; Cf, cephalothin; Cip, Ciprofloxacin; Ctx, cefotaxime; Cz, cefazolin; Fep, cefepime; Fox, cefoxitin; Gm, gentamicin; Imp, imipenem; K, kanamycin; Na, nalidixic acid; Nor, norfloxacin; S, streptomycin; Sxt, trimethoprim/sulfamethoxazole; Te, tetracycline.

In the present study, Na resistance (52.9%) was predominant among the 51 *Salmonella* isolates. Twenty-seven Na^R^ isolates were serotyped as *S.* Enteritidis (n = 18), *S.* Montevideo (n = 5), *S.* Senftenberg (n = 2), and *S.* Newport (n = 2). Na resistance does not seem to have decreased during the last several years in South Korea and rates of resistance remain higher compared to those reported in other countries. Na resistance rates among *Salmonella* strains originating from chickens were found to range from 37% to 94.3% over the last 6 years [8-12]. In contrast, *Salmonella* isolates from poultry meats from several sources in Canada were shown to lack Na resistance [13] while very low levels of resistance were observed among isolates from Japan (10.9%) [14] and the UK (28.2%) [15].

MICs for Na and Cip are presented in Table 2. Among the 51 isolates, 27 (52.9%) were Na^R^ (MIC ≥ 512 μg/mL) while the remaining 24 (47.1%) were susceptible to Na (MIC = 4–16 μg/mL). Interestingly, all 51 isolates were susceptible to Cip (MIC ≤ 0.5 μg/mL). In addition, all 18 *S.* Enteritidis isolates collected in the present study were highly resistant to Na (MIC ≥ 512 μg/mL) while all eight *S.* Typhimurium isolates were susceptible (MIC = 8–16 μg/mL).

**Table 2:** **Amino acid substitutions in *gyrA *** **and *parC *** **and antimicrobial resistance profiles in 51 *Salmonella *** **isolates from poultry slaughterhouses by nalidixic acid resistance**

**Resistance pattern of nalidixic acid**	**Serovars (No. of isolates )**	Amino acid substitution in ^*^		**MICs**^**‡ **^**(mg/l)**		**Antimicrobial resistance profiles**^**§**^
		**GyrA**	**ParC**	**Na**	**Cip**	
Resistant	Enteritis (18)	D87G	WT^†^	512	0.25	AmCSTe (1), AmS (2), STe (1)
				512	0.5	AmCfS (2), AmS (2)
		D87Y	WT	>512	0.5	- (8)
		D87N	WT	>512	0.5	AmCfCzGmKCazCtxFepSTe (1), - (1)
	Montevideo (5)	D87G	WT	>512	0.25	- (2)
				512	0.25	- (3)
	Senftenberg (2)	D87N	WT	512	0.5	- (1)
		D87G	WT	512	0.25	- (1)
	Newport (2)	D87Y	WT	512	0.5	- (1)
				>512	0.5	- (1)
Susceptible	Typhimurium (8)	WT	WT	8	<0.0625	- (6)
				8	0.25	- (1)
				16	<0.0625	STe (1)
	Montevideo (4)	WT	WT	4	<0.0625	GmK (2), - (2)
	Hadar (5)	WT	WT	4	<0.0625	AmCfCzSTe (1)
				8	<0.0625	AmCfCzKSTe (1), STe (2), KS (1)
	Ohio (3)	WT	WT	4	<0.0625	STe (1), - (2)
	London (3)	WT	WT	4	<0.0625	AmCfSTe (1)
				8	<0.0625	- (2)
	Hogton (1)	WT	WT	8	<0.0625	KSTe (1)

*D, aspartic acid; G, glycine; N, asparagine; Y, tyrosine.

^†^WT, wild type.

^‡^ Cip, ciprofloxacin; Na, nalidixic acid.

^§^Am, ampicillin; C, chloramphenicol; Caz, ceftazidime; Cf, cephalothin; Cz, cefazolin; Ctx, cefotaxime; Fep, cefepime; Gm, gentamicin; K, kanamycin; S, streptomycin; Te, tetracycline.

Table 2 shows amino acid substitutions in *gyrA* and *parC* found in the 27 Na^R^ isolates. These results indicate that mutations only at position 87 in *gyrA* conferred both Na resistance and Cip susceptibility. All 27 Na^R^ isolates possessed three different types of point mutations only at position 87 in *gyrA* and carried a D87 to G, Y, or N substitution. Among these 27 Na^R^ isolates, the most common *gyrA* mutation was D87G (51.9%) followed by D87Y (37.0%) and D87N (11.1%). No mutations at S83 of *gyrA* were observed in any isolate from the present study. No mutation in *parC* that affected resistance or susceptibility was identified. However, silent mutations at V67, H75, H77, V100, D101, and G102 were found within the QRDR of *parC*.

Our data may confirm that the mutations at S87 of *gyrA* alone can be sufficient to induce Na resistance and Cip susceptibility. Additionally, results from the present study imply that *parC* mutations are not necessary to obtain a high level of Na resistance as hypothesized in previous reports [16,17].

Our findings contrast with those generated by Griggs *et al.*[6] and Liebana *et al*. [18] showing that 95.4% and 50% of *Salmonella* isolates from animals contained a S83 mutation. Mutations at S83 of *gyrA* have been described in *Salmonella* isolates from humans and animals [6], and are thought to promote resistance to Na while reducing fluoroquinolone susceptibility.

Piddock [19] also reported that the number of quinolone-resistant *Salmonella spp.* encountered in human and veterinary medicine is increasing. Other previous investigations [20-22] demonstrated that a single mutation in the *gyrA* gene is associated with Cip susceptibility. In contrast, *parC* mutations can result in a high level of fluoroquinolone resistance [21].

## Conclusion

In conclusion, results from the present study showed that mutations in codon D87 of the *gyrA* gene are sufficient for conferring Na resistance and Cip susceptibility. Our findings also suggest that mutations in codon S83 of the *gyrA* gene are not a main factor of Na resistance, and *parC* mutations are not essential for quinolone resistance. Therefore, prudent use of antimicrobials and continued surveillance are absolutely needed to inhibit the increasing prevalence of quinolone resistance.

## Abbreviations

QRDR: Quinolone resistance-determining region; ISO: International standardization organization; CLSI: Clinical and laboratory standards institute; MICs: Minimal inhibition concentrations; BLAST: Basic local alignment search tool; NCBI: National center for siotechnology information.

## Competing interests

The authors declare that they have no competing interests.

## Authors’ contributions

DHB analyzed the samples, performed the statistical analysis and wrote the manuscript. HJB and SJJ helped collect and analyse the samples. YJL provided the basic format of the study, obtained the funding and acted as study team leader. All authors read and approved the final manuscript.
